# Supplementary material for: General framework for E(3)-equivariant neural network representation of density functional theory Hamiltonian
Source: Nat Commun. 2023 May 18;14:2848. doi: 10.1038/s41467-023-38468-8 (PMC10199065; doi:10.1038/s41467-023-38468-8)
Supplement: Supplementary file 1 — Supplementary information [file 41467_2023_38468_MOESM1_ESM.pdf]

Supplementary information for
General framework for E(3)-equivariant neural network representation of density functional theory Hamiltonian

Xiaoxun Gong,[1,2,*] He Li,[1,3,4,*] Nianlong Zou,[1] Runzhang Xu,[1] Wenhui Duan,[1,3,4,5,†] and Yong Xu[1,4,5,6,‡]

[1] *State Key Laboratory of Low Dimensional Quantum Physics and Department of Physics, Tsinghua University, Beijing, 100084, China*
[2] *School of Physics, Peking University, Beijing 100871, China*
[3] *Institute for Advanced Study, Tsinghua University, Beijing 100084, China*
[4] *Tencent Quantum Laboratory, Tencent, Shenzhen, Guangdong 518057, China*
[5] *Frontier Science Center for Quantum Information, Beijing, China*
[6] *RIKEN Center for Emergent Matter Science (CEMS), Wako, Saitama 351-0198, Japan*

Contents

Supplementary Note 1. Performance of DeepH-E3 on monolayer graphene and MoS_2 2

Supplementary Note 2. Comparison of VASP and OpenMX on studying twisted bilayer graphene 2

Supplementary Note 3. Detailed results of bilayer bismuthene and Bi_2Se_3 2

Supplementary Note 4. Network hyperparameters and their selection strategy 3

Supplementary Note 5. The use of E3LayerNorm 4

Supplementary References 6

* These authors contributed equally: Xiaoxun Gong and He Li.
† duanw@tsinghua.edu.cn
‡ yongxu@mail.tsinghua.edu.cn

Supplementary Note 1. Performance of DeepH-E3 on monolayer graphene and MoS_2

Detailed analysis of the performance of DeepH-E3 studying monolayer graphene and MoS_2 can be found in Supplementary Figure 1.

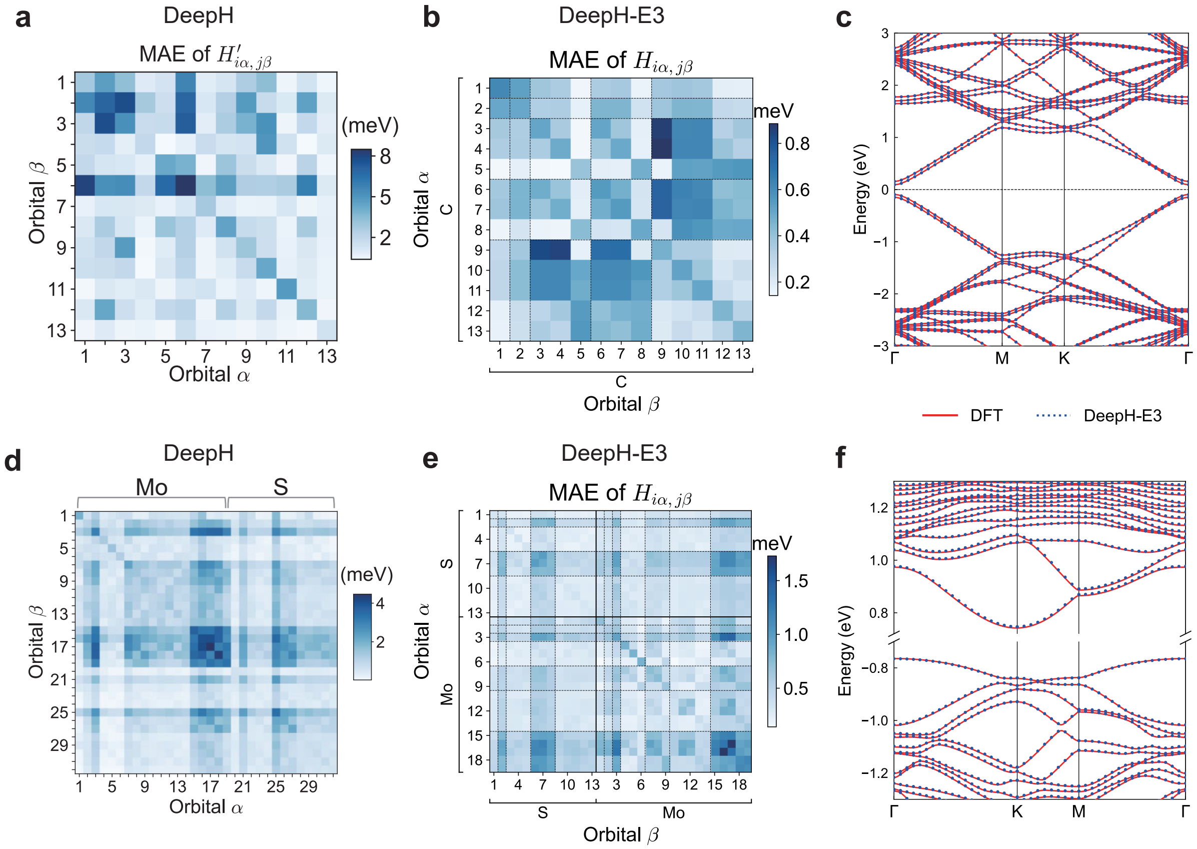

Supplementary Figure 1. Performance of DeepH-E3 studying monolayer graphene and MoS_2, compared to DeepH. (**a**,**b**) Mean absolute errors (MAEs) of DFT Hamiltonian matrix elements for different orbitals obtained by DeepH and DeepH-E3 studies of monolayer graphene. (**c**) Band structures of a perturbed graphene supercell computed by DeepH-E3 and DFT. (**d**-**f**) Results for monolayer MoS_2, similar to (**a**-**c**). The MAEs are averaged over all bonds and all structures in the test set. The band structures are calculated from a randomly selected structure from the test set. Source data are provided with this paper.

Supplementary Note 2. Comparison of VASP and OpenMX on studying twisted bilayer graphene

To check the influence of basis set and pseudopotential on DFT results, we calculated the electronic structure of a twisted bilayer graphene (twist angle $\theta = 6.01°$) by VASP and OpenMX as shown in Supplementary Figure 2. Our test calculations indicate that the use of different basis sets and pseudo potentials (VASP vs. OpenMX) has minor influence on the calculated electronic bands, especially those near the Fermi level, at least for this particular material. As the size of material system increases, the calculation gets more and more difficult to converge and the corresponding numerical error typically grows, which might enhance the band-structure discrepancies between the two approaches. This, however, is difficult to check for the magic-angle twisted bilayer graphene, because we cannot do the benchmark calculation directly by using the OpenMX code as limited by the huge computational cost.

Supplementary Note 3. Detailed results of bilayer bismuthene and Bi_2Se_3

To test the performance of DeepH-E3 on studying materials with strong SOC, we carry out experiments on bilayers of 2D materials Bi_2Se_3 and bismuthene. The averaged MAE of the Hamiltonian matrix on non-twisted bilayer Bi_2Se_3

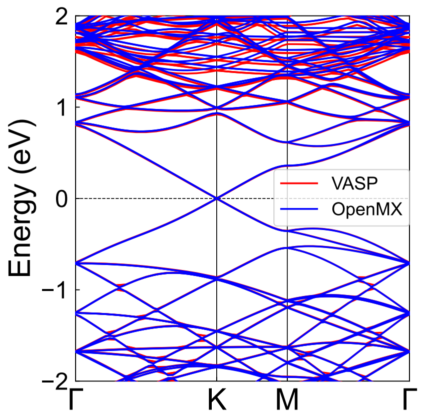

Supplementary Figure 2. Band structures of twisted bilayer graphene with twist angle $\theta = 6.01°$ calculated by VASP and OpenMX. Source data are provided with this paper.

and bismuthene test sets are as low as 0.42 meV and 0.26 meV, respectively. The network also has outstanding accuracy on the twisted structures. For twisted Bi_2Se_3 with twist angle $\theta = 13.2°$, the averaged MAE is 0.35 meV. For twisted bismuthene with $\theta = 7.34°$, the averaged MAE is 0.30 meV. The MAEs and predicted band structures are shown in Supplementary Figure 3.

To find the optimal model hyperparameter setup for practical twisted material study, a model with reduced number of parameters is also tested on the Bi_2Se_3 dataset. The results are summarized in Supplementary Table 1. We find that the accuracy of the reduced model is also sufficient for practical use, and the reduced model only takes 30 hours by one GPU for training.

Supplementary Table 1. Comparison of two models on studying nontwisted and twisted bilayer Bi_2Se_3. The model parameters are all real numbers, and mean absolute errors (MAEs) are in units of meV. [a]

Model	Training time	Number of parameters	MAE (nontwisted)	MAE ($\theta = 13.2°$)
Full	3d 10h	1.5×10^6	0.42	0.35
Reduced	1d 6h	5.9×10^5	0.79	0.63

[a] Both full and reduced models are trained on a single NVIDIA GeForce RTX 3090 GPU. The reduced model can reach sub-meV precision as well, which is remarkable because the model is required to fit 2.8×10^9 complex matrix elements.

Supplementary Note 4. Network hyperparameters and their selection strategy

Summary of the network hyperparameter setup for each material system can be found in Supplementary Table 2. In our experiments, we find that most of the hyperparameters have minor influence on the final model performance as long as they are within a reasonable range. We select the optimal hyperparameters by the following strategies. On the learning rate, we find that too large learning rate might cause instability in the training process: the loss function might suddenly blow up to a very high value. Too small learning rate will make the training slow and increase the probability of overfitting. Therefore, we first restrict the learning rate within a reasonable range to avoid these two situations, and then select the learning rate that gives the best model. On the batch size, it is usually limited by the computer memory. One single crystalline structure can have as many as $\sim 10^6$ Hamiltonian matrix elements, so the batch size is usually chosen to be 1. When studying relatively simple materials, the batch sizes will be increased in order to speed up the training process. On hyperparameters that are related to the expressive power of the model, such as the length of the internal vertex and edge features, the maximum angular momentum of spherical harmonics and the number of message-passing layers, there is a tradeoff between the network performance and the training time. Usually, more complex neural networks could realize smaller prediction error. We choose those parameters to make sure that we could produce the best results within an affordable training time.

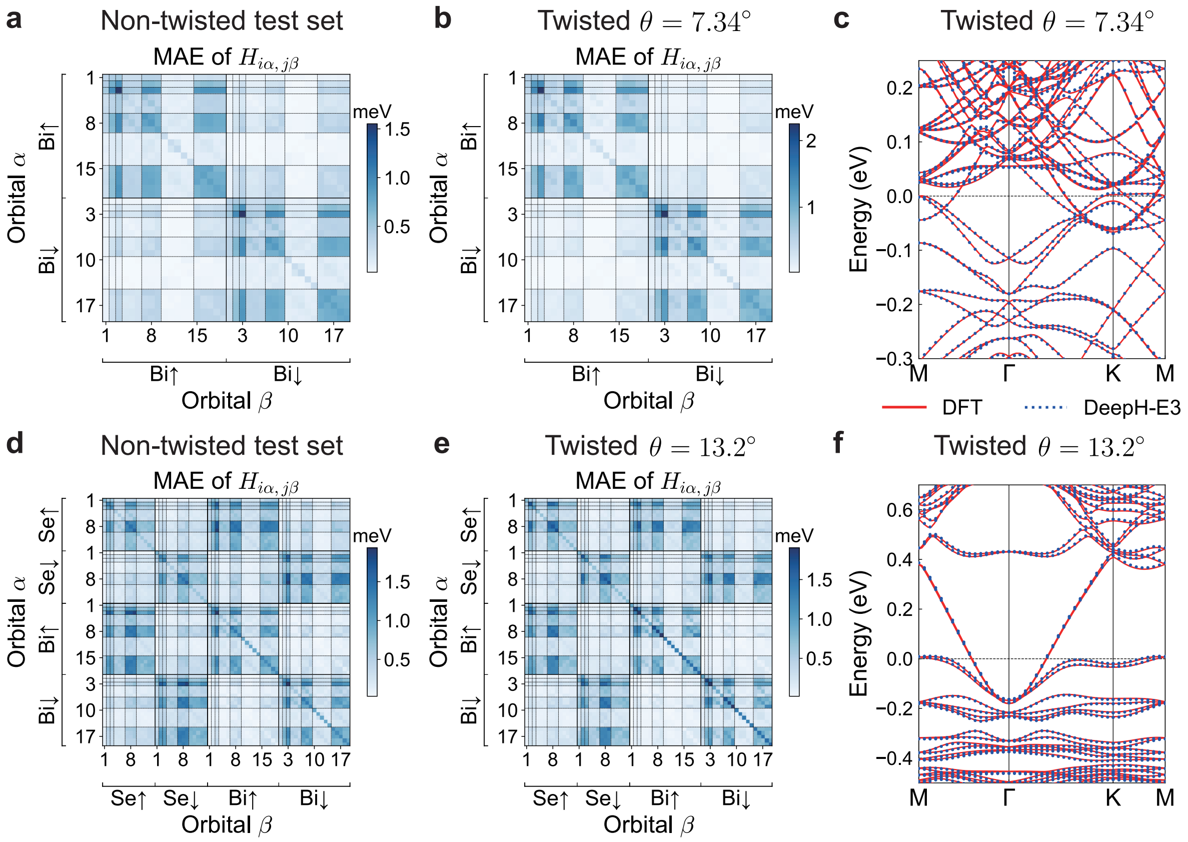

Supplementary Figure 3. Performance of DeepH-E3 on twisted bilayer bismuthene and Bi_2Se_3 . (**a**,**b**) Mean absolute errors (MAEs) of DFT Hamiltonian matrix elements for different orbitals obtained by DeepH-E3 studies of (**a**) non-twisted and (**b**) twisted bilayer bismuthene test sets. (**c**) Band structure of twisted bilayer bismuthene computed by DeepH-E3 and DFT. (**d**-**f**) Results for bilayer Bi_2Se_3, similar to (**a**-**c**). The MAEs are averaged over all bonds and all structures in the test set. The twist angle $\theta = 7.34°$ for twisted bilayer bismuthene and $\theta = 13.2°$ for twisted bilayer Bi_2Se_3. Source data are provided with this paper.

Supplementary Table 2. Summary of hyperparameter setup in the experiments discussed in this work. Here $l_{\max}$ is the maximum degree of spherical harmonics used. Each layer contains one vertex update block and one edge update block. `32x1o` denotes 32 vectors carrying the $l = 1$ representation with odd parity, similar for others. The networks are optimized using Adam algorithm with $\beta_1 = 0.9, \beta_2 = 0.999$. Models for bilayer Bi_2Te_3 with and without SOC used the same hyperparameters.

System	Number of structures			Intermediate edge and vertex features	$l_{\max}$	# layers	Learning rate	Batch
	Training	Validation	Test					
Monolayer graphene	270	90	90	`64x0e+32x1o+16x2e+8x3o+8x4e+4x5o`	5	3	0.003	1
Monolayer MoS_2	300	100	100	`64x0e+32x1o+16x2e+8x3o+8x4e+4x5o`	5	3	0.005	1
Bilayer graphene	180	60	60	`64x0e+32x1o+16x2e+8x3o+8x4e+4x5o`	5	3	0.003	1
Bilayer bismuthene	231	113	113	`64x0e+32x1o+16x2e+8x3o+8x4e+4x5o`	5	3	0.005	1
Bilayer Bi_2Se_3	231	113	113	`64x0e+32x1o+16x2e+8x3o+8x4e+4x5o`	5	3	0.005	1
Bilayer Bi_2Se_3 (reduced)	231	113	113	`32x0e+16x1o+8x2e+4x3o+4x4e`	4	3	0.002	1
Bilayer Bi_2Te_3	204	38	12	`64x0e+32x1o+16x2e+8x3o+8x4e`	5	3	0.004	2
Ethanol	25000	500	4500	`64x0e+32x1o+16x2e+8x3o+8x4e+4x5o`	5	4	0.005	300

Supplementary Note 5. The use of E3LayerNorm

Batch normalization [1] and layer normalization [2] are widely adopted techniques in machine learning to make the training process of neural networks efficient and stable. For DeepH-E3, batch normalization is not very helpful because every single crystalline material structure for training has a large amount of Hamiltonian matrix elements

and thus the batch size is usually chosen to be small. In this situation, it will be useful to develop a normalization scheme using the layer statistics while fully respecting the equivariance of the feature vectors. In our experiments, we find that the introduction of E3LayerNorm significantly stabilizes the training process (Supplementary Figure 4), thus enables higher learning rates, which is not only beneficial for the optimization of the neural network parameters but also improves the generalization ability of the model. In producing Supplementary Figure 4, we used the monolayer MoS_2 dataset for training, with 3 layers, starting learning rate 0.01, batch size 4, $l_{\max} = 4$ and intermediate feature vectors `64x0e+32x1o+16x2e+8x3o+8x4e`.

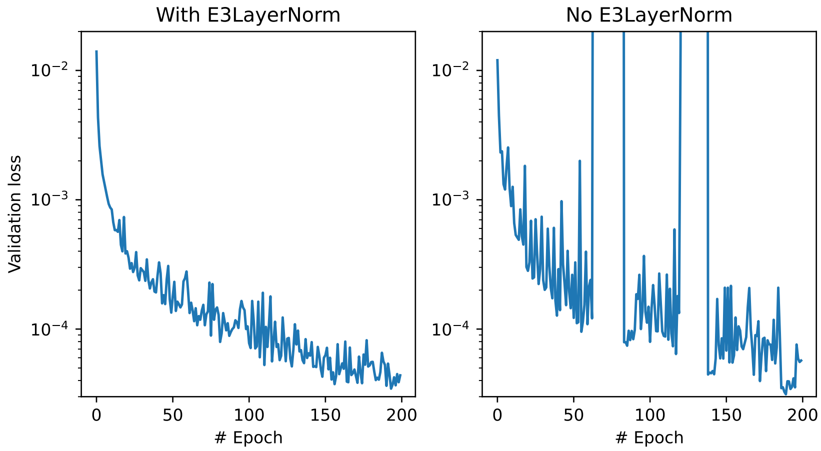

Supplementary Figure 4. Comparison of the training process with or without E3LayerNorm, using the monolayer MoS_2 dataset. The vertical axis measures the mean squared error of Hamiltonian matrix elements in unit of eV^2. When the loss blows up and does return to normal within 20 epochs, the model is automatically reverted to the state before with minimum loss, and learning rate is decreased by a factor of 0.8. Source data are provided with this paper.

Supplementary References

[1] S. Ioffe and C. Szegedy, Batch normalization: Accelerating deep network training by reducing internal covariate shift, in *International conference on machine learning* (PMLR, 2015) pp. 448–456.

[2] J. L. Ba, J. R. Kiros, and G. E. Hinton, Layer normalization, arXiv preprint arXiv:1607.06450 (2016).
